# Hemodialysis patients’ preferences for the management of anemia

**DOI:** 10.1186/s12882-017-0664-9

**Published:** 2017-07-28

**Authors:** Brett Hauber, John Caloyeras, Joshua Posner, Deborah Brommage, Spiros Tzivelekis, Allan Pollock

**Affiliations:** 1 RTI Health Solutions, 200 Park Offices Drive, Research Triangle Park, NC USA; 2 Amgen, Thousand Oaks, CA USA; 3 National Kidney Foundation, New York, NY USA

**Keywords:** Anemia, End-stage renal disease, Discrete-choice experiment, Conjoint analysis

## Abstract

**Background:**

Patient engagement in end-stage renal disease (ESRD) is expected to result in a more patient-centered approach to care that aligns with patients’ values, preferences, and goals for treatment. Nevertheless, no previous studies of which we are aware have evaluated patients’ benefit-risk preferences for the management of anemia associated with ESRD. The primary objective of this study was to quantify the tradeoffs patients are willing to make between cardiovascular risks associated with some anemia medicines and red blood cell (RBC) transfusions. A secondary objective was to quantify the importance of avoiding transfusion-related risks.

**Methods:**

A survey instrument was developed from the clinical literature, clinician input, patient-education resources, and a patient focus group. The survey instrument was qualitatively pretested before its administration to a broader sample of patients. The National Kidney Foundation invited individuals in the United States to participate in the survey. In a discrete-choice experiment (DCE), respondents chose between two hypothetical anemia medications in a series of questions. Each medication was defined by symptom relief, frequency of transfusions, cardiovascular risk, mode of administration, and out-of-pocket cost. The survey also included a best-worst scaling (BWS) exercise to quantify the importance of avoiding attributes of blood transfusions. Results from the DCE were used to estimate relative importance and marginal willingness to pay. Results from the BWS were converted to relative importance weights.

**Results:**

A total of 200 individuals completed the survey. Patients were willing to accept a 6% medication-related risk of heart attack to avoid having two RBC transfusions per month. Symptom relief and mode of administration were of moderate importance. The most important transfusion-related risk to avoid was transfusion-related lung injury.

**Conclusions:**

Patients with ESRD and anemia have measurable treatment preferences and are willing to accept risks associated with anemia medications to avoid transfusions.

**Electronic supplementary material:**

The online version of this article (doi:10.1186/s12882-017-0664-9) contains supplementary material, which is available to authorized users.

## Background

Anemia is prevalent among patients with end-stage renal disease (ESRD) and, left untreated, has a negative impact on quality of life [1–3]. There are important differences among the treatment options for anemia. Iron or erythropoietin-stimulating agents (ESAs) may be administered to increase hemoglobin levels [2]. Severe cases of anemia in patients with ESRD undergoing hemodialysis may be treated with both ESAs and red blood cell (RBC) transfusions to manage hemoglobin levels. ESAs decrease the need for RBC transfusions, thereby improving patients’ health-related quality of life, but carry risks of cardiovascular events, including stroke, and increased mortality [2, 4, 5]. RBC transfusions are used in acute situations and in patients who do not respond to ESAs but are associated with uncommon risks such as acute lung injury, circulatory overload, and allosensitization to HLA antigens, which may interfere with subsequent kidney transplant [2]. The Kidney Disease Improving Global Outcomes (KDIGO) clinical practice guideline for anemia in chronic kidney disease recommends avoiding RBC transfusions for patients eligible for transplants to minimize the risk of allosensitization [2].

In serious chronic diseases such as ESRD, patient-centered care is essential to optimizing outcomes and patients’ satisfaction with treatment [6]. A tenet of patient-centered care is shared decision making, whereby treatment decisions incorporate not only clinical evidence but also patients’ values, priorities, and preferences [7]. For anemia management, health care providers should help patients understand the risks and benefits of available treatment options (such as iron supplementation, ESAs, or RBC transfusion), align treatment decisions with what matters most to individual patients, and promote patient safety [7, 8].

Previous preference work in anemia related to ESRD has focused on the clinical tradeoffs affecting providers’ decisions to transfuse patients undergoing hemodialysis [9], but, to our knowledge, no previous studies have evaluated hemodialysis patients’ benefit-risk preferences for the management of anemia. Therefore, the primary objective of this study was to elicit patients’ preferences for features of anemia therapy, including the potential effect of treatment on anemia symptoms, the frequency of blood transfusions, cardiovascular risk, and mode of administration. A secondary objective was to quantify the relative importance of avoiding potential risks of transfusions to understand the extent to which and the reasons why transfusions may be an undesirable treatment option from the patients’ perspective. This study was part of a larger effort to engage ESRD patients on their preferences, and we conducted a study using similar methods to evaluate patients’ preferences for the management of secondary hyperparathyroidism [10].

## Methods

To elicit patients’ preferences for anemia-management options, we developed and administered a discrete-choice experiment (DCE) survey in which patients chose between hypothetical treatment options defined by attributes to which different pre-specified levels were applied (see Table 1). By analyzing the pattern of responses to a series of DCE questions, one may infer the tradeoffs patients would be willing to make among treatment attributes and may quantify conditional relative importance (i.e., the importance of each attribute, conditional upon the levels of that attribute, relative to all other attributes). When out-of-pocket cost is included as an attribute in the survey, the results can be expressed as the value patients place on individual changes in treatment outcomes or processes [11]. This value is sometimes referred to as *marginal willingness to pay*.

**Table 1:** Attributes and levels in the treatment-choice questions

Attribute	Levels
Chance that the medicine makes you feel better by relieving your anemia symptoms	▪ 75 out of 100 (75%)
	▪ 50 out of 100 (50%)
	▪ 25 out of 100 (25%)
Number of red blood cell transfusions needed each month	▪ 0 transfusions each month
	▪ 1 transfusion each month
	▪ 2 transfusions each month
Risk of dying from a heart attack or stroke because of the medicine	▪ 0 out of 100 (0%)
	▪ 2 out of 100 (2%)
	▪ 6 out of 100 (6%)
How you receive the medicine	▪ An injection directly into the dialysis line
	▪ An injection under your skin
Out-of-pocket cost each month	▪ $50 each month
	▪ $100 each month
	▪ $200 each month

Note: The survey instrument in the Additional file 1 provides additional details about how the attributes and levels were presented

### Survey instrument development

Developing the DCE survey instrument was a five-step process, involving a review of product labels, solicitation of clinician input, a review of the National Kidney Foundation’s (NKF’s) patient-education materials, conduct of a patient focus group to inform selection of the attributes, and conduct of qualitative pretest interviews to ensure that the survey questions were clear and comprehensible and identify any refinements necessary before administration of the survey. Preliminary selection of the potentially relevant anemia attributes which were further refined in the focus group was based on product labels, the NKF’s patient-education materials, and input from nephrologists. The Additional file 1 provides additional details about both the focus group and qualitative pretests and presents the final survey instrument.

The DCE survey instrument, consistent with good research practices [12], was developed to elicit respondents’ preferences for two hypothetical medication options in a series of questions. The hypothetical treatments were defined by attributes of efficacy, safety, mode of administration, and out-of-pocket cost. The attributes and levels, defined in Table 1, were chosen to represent the features of disease management that are relevant and important to patients as well as the features of existing anemia-management options. The levels of each attribute included in the survey were chosen to encompass the range of clinically relevant levels of that attribute and to reflect the extent to which patients were willing to trade off changes in the level of that attribute with changes in the levels of other attributes. Out-of-pocket cost was included to allow the estimation of marginal willingness to pay for changes in treatment features.

Before the DCE and BWS questions were presented to participants, each attribute was described to participants using clinically accurate, yet patient-friendly language. Probabilities were described as ratios, percentages, and using pictograms. Participants were presented with information about how to interpret probabilities and asked to complete 2 questions to assess their comprehension of the probability information. If a respondent answered one of these questions incorrectly, the information about interpreting probabilities was repeated to assist the participant in understanding the probabilities. This approach is consistent with that used in other DCE studies [13].

**Fig. 1:**
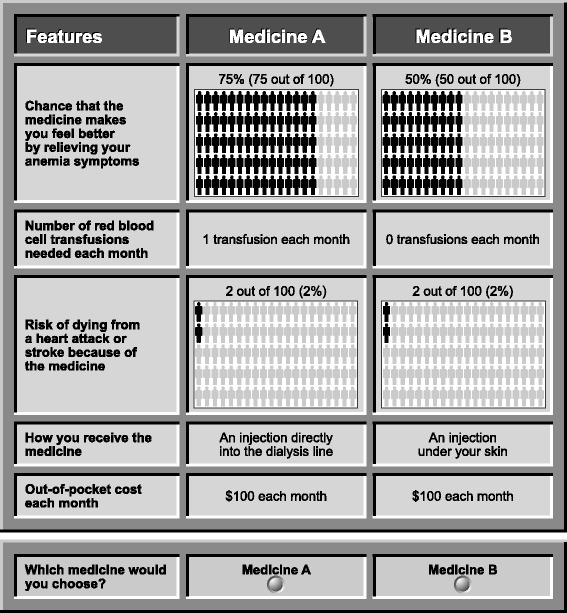
Example of a Treatment-Choice Question

**Fig. 2:**
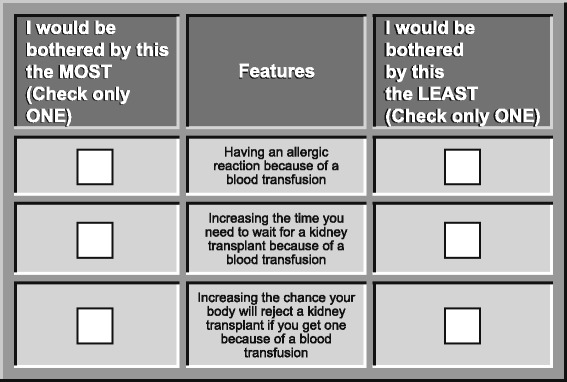
Example of a Best-Worst Scaling Question

In each treatment-choice question, respondents chose between the two medication alternatives. Figure 1 presents an example of a treatment-choice question included in the survey instrument. A best-worst scaling (BWS) exercise was included in the study to quantify the relative importance patients place on avoiding potential risks of transfusions. The items in the BWS exercise included six attributes potentially associated with blood transfusions and one attribute potentially associated with ESAs; the attributes are presented in Table 2. In each BWS question, patients were presented with a list of three treatment attributes and asked to choose which attribute would be most and least bothersome. Figure 2 presents an example BWS question. In addition, the survey instrument included demographic questions and disease history and treatment questions.

**Table 2:** Attributes included in the best-worst scaling questions

Attributes	
Having an allergic reaction because of a blood transfusion	
Having lung damage because of a blood transfusion that makes it hard to breathe	
Getting a serious infection because of a blood transfusion	
Increasing the time you need to wait for a kidney transplant because of a blood transfusion	
Increasing the chance your body will reject a kidney transplant if you get one because of a blood transfusion	
Needing to arrange transportation and spend 1 to 2 h at a hospital or infusion center to receive a blood transfusion	
Having a 1% risk of dying from a heart attack or stroke because of the anemia medicine

To prepare the survey for online administration, experimental designs for both the DCE and BWS exercises were developed following good research practices [14]. The final DCE experimental design included 40 questions divided into 5 blocks, each with 8 questions. Each respondent was randomly assigned to answer the choice questions in one block. The pattern of responses to such a series of DCE questions provided information that was used to estimate the extent to which changes in the levels of treatment attributes affected treatment choice. The final BWS experimental design included seven questions with three items in each question. All respondents answered the same set of seven BWS questions.

### Study population

The target sample for this study was 200 participants. Because there is no analytic solution to determine minimum sample size in DCE surveys without knowing the preference weights in advance, we relied on a review of existing empirical studies, which indicated that a sample of 200 patients is likely sufficient to estimate the preference weights of interest in a DCE with the number of attributes and levels included in this study [15]. Individuals in the United States registered as patients in NKF’s opt-in member database, maintained primarily for communications, were invited to be screened for eligibility for the study through an e-mail invitation that explained the study. To be eligible for participation, respondents were required to be aged 18 years or older, to have self-reported ESRD, and to be receiving in-center hemodialysis. Respondents were not required to have anemia in order to complete the survey. All study participants provided informed consent. Each participant who completed the survey was provided with a $25 gift card as compensation for his or her time and effort for completing the survey. The study was approved by the Office of Research Protection and Ethics at RTI International and complied with the Declaration of Helsinki.

### Statistical analyses

#### Discrete-choice experiment analyses

The DCE data were analyzed using a random-parameters logit model following good research practices [16]. This model yielded a relative preference parameter for each attribute level included in Table 1. The parameter estimates from a random-parameters logit model can be interpreted as preference weights indicating the relative strength of preference for each attribute level.

With the exception of those for out-of-pocket cost, the levels of all attributes were modeled as categorical, effects-coded variables [16, 17]. Out-of-pocket cost of the medicine was modeled as an interaction between the cost level shown for the medicine and the natural log of the respondent’s reported household income in the previous calendar year. To determine the conditional relative importance of an attribute, the difference in preference weights between the attribute level with the highest preference weight and the level with the lowest preference weight was calculated. Marginal willingness to pay is the change in out-of-pocket cost per month that respondents would accept in exchange for a given improvement in treatment outcomes. It was calculated as the increase in out-of-pocket cost per month that would exactly offset the perceived benefit of given improvements in each attribute. Ninety-five percent confidence intervals (CIs) were calculated for all estimated preference weights, conditional relative importance estimates, and marginal willingness-to-pay estimates.

### Best-worst scaling analyses

The relative bother of each transfusion feature was estimated using a random-parameters logit model. Relative bother estimates reflect the relative weights respondents placed on each item when selecting the most and least bothersome items. Larger coefficients indicate that the item was more bothersome. Conversely, smaller coefficients indicate that the item was less bothersome. To calculate estimates of relative importance of avoiding each transfusion feature from the estimates of relative bother, we used a probability-based rescaling procedure [18].

## Results

### Respondent characteristics

A total of 7489 individuals were invited to be screened for eligibility for the study through an e-mail invitation, and 657 individuals accessed the survey. Of those who accessed the survey, 233 (5% of those who were invited and 35% of those who accessed the survey) were eligible. Of those who were eligible, 219 (94%) consented to participate. Of those who consented to participate, 200 (91%) completed the survey. Table 3 presents a summary of respondents’ demographic and clinical characteristics.

**Table 3:** Respondents’ characteristics

Characteristic	Statistic or category	Overall (*N* = 200)
*Demographic characteristics*		
Age (years)	Mean (SD)	54.1 (13.4)
	Median	54.0
Gender	Female	98 (49.2%)
	Male	99 (49.7%)
	Prefer not to answer	2 (1.0%)
	Missing	1
Race/ethnicity	White or Caucasian	102 (51.0%)
	Black or African American	66 (33.0%)
	Other	36 (18.0%)
	Prefer not to answer	4 (2.0%)
Highest level of education	High school or less than high school	44 (22.0%)
	More than high school	156 (78.0%)
*Clinical characteristics*		
Duration of time receiving dialysis	Less than 6 months	3 (1.5%)
	6 months to less than 1 year	8 (4.0%)
	1 year to less than 2 years	30 (15.0%)
	2 years to less than 5 years	89 (44.5%)
	5 years to less than 10 years	54 (27.0%)
	10 years or more	16 (8.0%)
Ever received a diagnosis of anemia from a doctor or health care professional	Yes	163 (81.5%)
	No	21 (10.5%)
	Don’t know/not sure	16 (8.0%)
Ever experienced the following anemia symptoms (check all that apply)	Feel tired and have little energy for your daily activities	152 (93.3%)
	Have a rapid heartbeat	50 (30.7%)
	Have little or no appetite	80 (49.1%)
	Feel depressed or “down in the dumps”	82 (50.3%)
	Have trouble thinking clearly	69 (42.3%)
	Feel dizzy or have headaches	71 (43.6%)
	Feel short of breath	76 (46.6%)
	Have trouble sleeping	113 (69.3%)
	Look pale	48 (29.4%)
	None of the above	3 (1.8%)
Which of the following anemia symptoms would be most bothersome?	Feeling tired and having little energy for your daily activities	125 (62.5%)
	Shortness of breath	51 (25.5%)
	Trouble thinking clearly	24 (12.0%)
Ever had a red blood cell transfusion	Yes	90 (45.0%)
	No	92 (46.0%)
	Don’t know/not sure	18 (9.0%)
Ever experienced a heart attack or stroke	Yes	40 (20.0%)
	No	155 (77.5%)
	Don’t know/not sure	5 (2.5%)
Usual mode of receiving anemia medication^a^	As an injection directly into the dialysis line during my regular dialysis treatment	128 (64.0%)
	As an injection under my skin while I am receiving my regular dialysis treatment	7 (3.5%)
	I do not currently take an anemia medicine	65 (32.5%)

SD = standard deviation

Note: Percentages do not include missing responses in the denominator

^a^Of the 135 respondents who reported taking an anemia medicine, 123 respondents have or have had anemia, 5 respondents have never had anemia, and 7 respondents were not sure if they have or have had anemia

### Preference weights and relative importance of treatment attributes

**Fig. 3:**
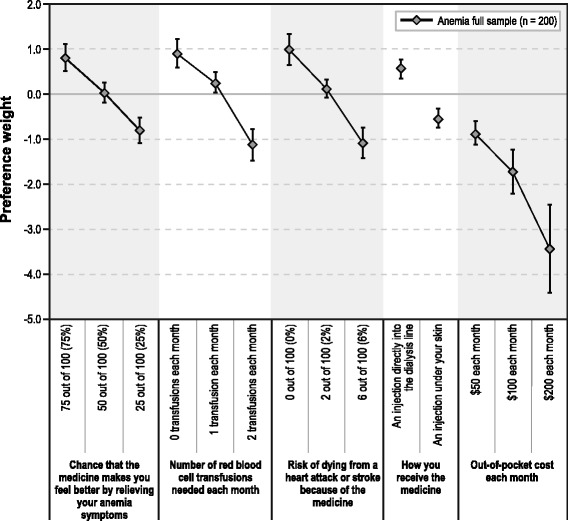
Anemia Treatment Preference Weights

Figure 3 presents the preference weights indicating the relative strength of preference for each attribute level. More preferred outcomes have higher preference weights. For example, 75% was preferred to 50% for *chance that the medicine makes you feel better by relieving your anemia symptoms*, and 50% was preferred to 25%. Similarly, zero transfusions for *number of red blood cell transfusions needed each month* was preferred to one transfusion, which was preferred to two transfusions. In fact, the estimated preference weights for better clinical outcomes (e.g., greater efficacy, fewer transfusions, lower risk, and lower cost) were consistently higher than those for worse clinical outcomes across all attributes. The vertical distance between preference weights within an attribute represents the relative importance of moving from one level to another of that attribute. For example, the change from two transfusions to one transfusion had a relative importance of 1.39 (= 0.248 – [−1.139]) (95% CI, 0.92–1.85). The change from one transfusion to zero transfusions had a relative importance of 0.64 (= 0.891–0.248) (95% CI, 0.25–1.04). Therefore, the change from two transfusions to one transfusion was approximately 2.2 (1.39 ÷ 0.64) times as important as a change from one transfusion to zero transfusions.

Comparing across attributes, the relative importance of the change in *risk of dying from a heart attack or stroke because of the medicine* from 6% to 2% had a relative importance of 1.20 (= 0.114 – [−1.087]) (95% CI, 0.77–1.63), whereas the change in *chance that the medicine makes you feel better by relieving your anemia symptoms* from 25% to 50% had a relative importance of 0.83 (= 0.016 – [−0.818]) (95% CI, 0.46–1.20), and the change in *number of red blood cell transfusions needed each month* from two transfusions to one transfusion had a relative importance of 1.39, as mentioned previously. Therefore, the change in *risk of dying from a heart attack or stroke because of the medicine* from 6% to 2% was approximately 1.4 (= 1.20 ÷ 0.83) times as important as the change in *chance that the medicine relieves your anemia symptoms* from 25% to 50% and 0.9 (= 1.20 ÷ 1.39) times as important as the change in *number of red blood cell transfusions needed each month* from two transfusions to one transfusion.

The difference in the preference weights of the most preferred and least preferred level of an attribute is a measure of the conditional relative importance of the attribute over the range of levels included in the study. *Out-of-pocket cost *had the greatest conditional relative importance (2.59 [95% CI, 1.87–3.31]), and the conditional relative importance of the *risk of dying from a heart attack or stroke* and *number of RBC transfusions needed *were similar (2.06 [95% CI, 1.44–2.68] and 2.03 [95% CI, 1.44–2.62], respectively). The *chance that the medicine makes you feel better by relieving your anemia symptoms* attribute had a conditional relative importance of 1.62 (95% CI, 1.09–2.15), and the *how you receive the medicine* attribute had a conditional relative importance of 1.08 (95% CI, 0.69–1.47).

### Willingness to pay

Table 4 presents marginal willingness-to-pay estimates for improvements between adjacent levels of each attribute included in the study. To move from two RBC transfusions needed each month to zero transfusions, respondents were willing to pay $118 more per month (95% CI, $83–$152). To move from an injection under the skin to an injection directly into the dialysis line, respondents were willing to pay $63 more per month (95% CI, $41–$84).

**Table 4:** Marginal willingness-to-pay estimates for improvements in anemia treatment features (*N* = 200)

Attribute	Improvement	Willingness to pay per month (95% Confidence Interval)
Chance that the medicine makes you feel better by relieving your anemia symptoms	From 50% to 75%	$46 ($21–$70)
	From 25% to 50%	$48 ($25–$71)
	From 25% to 75%	$94 ($60–$128)
Number of red blood cell transfusions needed each month	From 1 transfusion to 0 transfusions	$37 ($14–$60)
	From 2 transfusions to 1 transfusion	$80 ($53–$108)
	From 2 transfusions to 0 transfusions	$118 ($83–$152)
Risk of dying from a heart attack or stroke because of the medicine	From 2% to 0%	$50 ($25–$74)
	From 6% to 2%	$70 ($46–$93)
	From 6% to 0%	$119 ($85–$154)
How you receive the medicine	From an injection under your skin to an injection directly into the dialysis line	$63 ($41–$84)

### Best-worst scaling

**Fig. 4:**
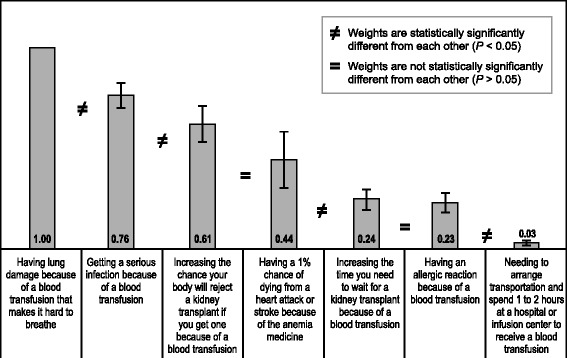
Best-Worst Scaling Relative Importance Estimates (*N* = 200)

Figure 4 summarizes the bother estimates for the treatment attributes included in the BWS questions. The most important transfusion feature to avoid was *having lung damage because of a blood transfusion that makes it hard to breathe*. The least important feature to avoid was *needing to arrange transportation and spend 1 to 2 h at a hospital or infusion center to receive a blood transfusion*.

## Discussion

This study sought to evaluate patients’ preferences for the attributes of available anemia treatments. We discovered that patients had discernible preferences among attributes and that they were willing to make tradeoffs among them, suggesting that they understood the benefits and risks of the hypothetical treatments included in the survey. Specifically, patients placed nearly equal value on avoiding two transfusions each month and eliminating a 6% medication-related risk of heart attack. To increase the probability of feeling better because of a medication from 25% to 75%, patients would be willing to accept approximately 1.7 transfusions per month or a 4.5% risk of medication-related heart attack. Interestingly, patients valued changing from a subcutaneous injection to having the injection directly in the dialysis line more than they valued eliminating one transfusion per month. The most important feature of transfusions to avoid was the risk of acute lung injury, which is the leading cause of transfusion-related mortality in the United States and which is a more common adverse event than transfusion-related infections [19, 20].

The primary means of eliciting input from patients with ESRD undergoing hemodialysis in the US is the In-Center Hemodialysis Consumer Assessment of Healthcare Providers and Systems (ICH-CAHPS) survey, in which the Centers for Medicare and Medicaid Services invite individuals who receive hemodialysis at participating centers to complete a survey evaluating their perceptions of the quality of their care [21]. Participation in the survey program is requisite for facilities providing in-center dialysis for 30 or more survey-eligible patients [22]. The survey includes a composite measure of how well dialysis providers provide information to patients, with an item specifically evaluating whether patients have been as involved as they wanted to be in choosing the treatment that is right for them in the last year [23]. However, the survey does not capture patients’ thinking about or specific preferences for the management of ESRD and its complications, including anemia. As health care providers partner with patients to make treatment decisions for ESRD-related anemia, they must educate patients about the benefits and risks of the treatment options available and consider individual patients’ preferences, needs, and goals for treatment [7]. Our results suggest that physicians can engage patients in discussions of anemia management. Understanding patients’ preferences for anemia treatments, including transfusions and treatments to reduce the need for transfusion, and patients’ willingness to trade off among the benefits and risks of these treatments may help guide providers toward care that better aligns with patients’ preferences.

This study has several limitations that are common to survey and preference studies. The study population was a convenience sample recruited from an opt-in database of NKF members and is not necessarily representative of the broader population undergoing hemodialysis and experiencing anemia. Because patients were recruited through an opt-in database that does not contain patient demographic information, it is not possible to determine the extent to which patients who completed the survey may differ from those who were invited to participate but did not complete the survey. Further, the respondents’ engagement and health literacy may not represent those of the wider population. The study results may not be broadly generalizable and are subject to voluntary response bias, given that a relatively small proportion of invited participants accessed the survey. A comparison of the characteristics of survey respondents and those who declined to participate, to explore any potentially important differences, was not feasible. Nevertheless, the survey respondents were generally comparable with individuals in the US undergoing dialysis in terms of most demographic characteristics, including age, gender, and race/ethnicity [24].

In addition, respondents were asked to evaluate hypothetical treatments, and not all attributes of anemia treatments were included. As such, the results relate only to those attributes included in the survey. Finally, it should be noted that study data represent average preferences among study respondents, and individual patients’ specific preferences will vary and must be considered in clinical decision making.

## Conclusions

We found that patients who are undergoing hemodialysis understand anemia and have clear and measurable preferences for anemia management. Our findings suggest that anemia medications administered directly into the dialysis line to manage anemia symptoms and reduce the frequency of RBC transfusions are desirable to patients undergoing hemodialysis.

## Additional file

Additional file 1: Survey-development materials. (DOCX 194 kb)

## Abbreviations

BWS: Best-worst scaling
CI: Confidence interval
DCE: Discrete-choice experiment
ESA: Erythropoietin-stimulating agent
ESRD: End-stage renal disease
ICH-CAHPS: In-center hemodialysis consumer assessment of healthcare providers and systems
KDIGO: Kidney disease improving global outcomes
NKF: National Kidney Foundation
RBC: Red blood cell
