# Supplementary material for: Hemodialysis patients’ preferences for the management of anemia
Source: BMC Nephrol. 2017 Jul 28;18:253. doi: 10.1186/s12882-017-0664-9 (PMC5532766; doi:10.1186/s12882-017-0664-9)
Supplement: Additional file 1: — Survey-development materials. (DOCX 194 kb) [file 12882_2017_664_MOESM1_ESM.docx]

# additional file

# DEVELOPMENT OF THE SURVEY INSTRUMENT

Focus Group

To evaluate patients’ perceptions of anemia treatments and to further inform selection of the attributes for the survey instrument, a focus group was convened with patients with anemia on hemodialysis, who were recruited by a qualitative research facility in Raleigh, NC. Individuals were eligible to participate in the focus groups if they were 18 years of age or older, currently undergoing dialysis, and had a self-reported physician diagnosis of anemia.

Participants reported in writing the three most favorable and the three least favorable attributes of their anemia treatment and the one attribute they would change about their current treatment. Participants’ (deidentified) responses were displayed for a group discussion, after which participants took part in a ranking exercise to identify the five best and the five worst attributes from a set of attributes identified by the participants or hypothesized to be of interest. These steps confirmed that no important treatment attributes were missing and that patients understood the attributes.

Pretest Interviews

Before online administration, the survey instrument was qualitatively pretested in face-to-face interviews with a convenience sample of 10 patients with ESRD and anemia undergoing hemodialysis, recruited by a qualitative research facility in Raleigh, NC. Interviews were led by a member of the research team (BH) with extensive experience in conducting such interviews. During the pretest interviews, respondents were asked to think aloud as they completed the draft survey. While completing the survey in this manner, respondents were asked a series of debriefing questions to determine whether they understood the definitions and instructions, accepted the hypothetical context of the survey, and successfully completed the choice questions in the survey instrument as instructed.

Because the patients who participated in pretest interviews constituted a convenience sample, representativeness of the overall ESRD population cannot be ensured. The purpose of the pretest interviews was to elicit respondents’ reactions to the draft survey instrument and to determine which elements of the survey instrument required clarification or revision. The survey instrument was refined based on the results of the pretest interviews.

A comparison of the convenience sample of patients participating in the pretest interviews and the final analysis sample revealed the following differences:

- The pretest interview sample was younger on average than the analysis sample (47 years old vs. 54 years old)
- The pretest interview sample had a higher proportion of female participants than the analysis sample (70% vs. 49%)
- The pretest interview sample had a higher proportion of Black or African American participants than the analysis sample (80% vs. 33%)
- The pretest interview sample had a higher proportion of married participants than the analysis sample (60% vs. 44%)
- The pretest interview sample had a similar proportion of participants with a high school education or less to the analysis sample (20% vs. 22%)
- The pretest interview sample had a higher proportion of participants who were disabled or unable to work than the analysis sample (60% vs. 47%)
- The pretest interview sample had a similar proportion of participants on Medicare to the analysis sample (80% vs. 82%)
- The pretest interview sample had a similar proportion of participants with an annual income of less than $30,000 than the analysis sample (40% vs. 45%)

# Focus Group Discussion Guide

Introduction (~10 minutes)

**[Introduce moderator and note-taker. Review consent form, which will explain the purpose and format of the interview.]**

We are working with a biotechnology company to learn more about patient experiences with treatments for anemia in end-stage renal disease (ESRD). Anemia is a condition in which the body does not have enough healthy red blood cells. Red blood cells provide oxygen to body tissues. Treatments for anemia may include oral medicines, intravenous medicines, or blood transfusions.

Today we will ask you some questions about

- Your ***experiences*** with anemia treatments,
- What things you ***like and dislike*** about these treatments, and
- What you ***would change*** about these treatments if you could.

Please keep in mind that there are no wrong answers, so please share your opinions freely. You are the experts today, and we want to learn from you and your experiences.

During the focus group, we ask that you use only your first name, and please do not share any personal information such as your age, your doctor’s name, or where you live. We will not be recording any of this information in our notes. Your privacy is important to us, and we want to be extra careful that no one shares or feels obligated to share personal information unnecessarily.

**[Ask participants to read the informed consent form. Answer any questions regarding the informed consent form. Ask participants to sign the informed consent form. Then collect the signed informed consent forms and make sure that each participant has a copy of the informed consent form to keep.]**

**[Assign a participant number to each participant based on his or her position around the table starting with the first participant sitting to the left of the moderator and moving clockwise around the table. This participant number is for the research team’s use to be able to match the results from the index card feedback to the corresponding participant and will not be disclosed to the participant.]**

Treatment History (~10 minutes)

First, we’d like to learn a little more about your experiences with anemia and its treatment.

To begin with, will each of you tell me and the group

- When did you first start dialysis?
- When was the first time you were diagnosed with anemia?
- What types of treatments you have taken for anemia?
- How many of you have taken only one type of treatment since you were diagnosed? How many of you have taken more than one type of treatment? Can you tell me a little bit about the differences you noticed with the different types of treatments? **[Facilitator to probe regarding first and second lines of treatment and when new treatments were initiated versus participants experiencing only one type of treatment at various times.]**

We do not need to know the names of specific treatments. We are more interested in the types of treatments you have taken.

**[If no participants have mentioned intravenous medicines or blood transfusions, ask participants if anyone has taken an intravenous medicine or had a blood transfusion to treat anemia.]**

**[Ask all participants if their doctor has spoken to them about blood transfusions as a possible treatment for anemia.]**

Treatment Likes and Dislikes (~20 minutes)

Today, we want to learn more about your experiences and factors that contribute to your satisfaction with anemia treatments.

**[Hand out three cards to each participant. Each card should have the participant number listed at the top of the card.]**

Each of you has three cards.

On one card, please list the three things you ***like most*** about your current treatment. Please try to list three things; however, if you cannot think of three things, that is okay.

**[Collect the card containing the likes from each participant.]**

On the second card, please list the three things you ***dislike most*** about your current treatment. Please try to list three things; however, if you cannot think of three things, that is okay.

**[Collect the card containing the dislikes from each participant.]**

**[Write the likes on the flip chart (duplicates from multiple participants should not be written twice; however, the number of participants providing the same response can be indicated next to the response). When writing the items on the flip chart, ask for clarification if the response is not specific enough. For example, if the card states, “treatment works well,” ask the group what “works well” means.]**

**[Write the dislikes on the flip chart (duplicates from multiple participants should not be written twice; however, the number of participants providing the same response can be indicated next to the response). Again, please ask for clarification or specification if items are general or vague.]**

**[Ask participants if there are any features not listed on the flip chart that they feel should be listed.]**

Unmet Needs (~10 minutes)

On the third card, please write down your response to the following question:

- If there was one thing you **could change** about your current treatment, what would it be?

**[After everyone has completed his or her card…]**

We would now like you to share with the group the thing you would like to change about your current treatment.

**[Ask each participant to tell the group their answer. Write down answers on a flip chart (duplicates from multiple participants should not be written twice; however, the number of participants providing the same response can be indicated next to the response). Again, please ask for clarification or specification if items are general or vague.]**

# Break (~10 minutes)

**[During the break, enter 12 to 20 of the best and worst items into the best-worst scaling spreadsheet. Items in the best-worst scaling spreadsheet will include items raised by the focus group participants in the previous exercises in addition to items identified by the project team prior to the focus groups.]**

Best-Worst Scaling Exercise (~20 minutes)

Thank you all for sharing your opinions about your current anemia treatment experience and what you think could be better. We have one more exercise we would like you to help us with.

**[Hand out the best-worst scaling worksheet to each participant. Each worksheet should include the participant’s identification number.]**

We will now show you a few slides. On each slide, you will see five features of anemia treatments. Each feature has a number associated with it.

**[Show the first best-worst scaling slide.]**

In front of you there is a piece of paper with slide numbers listed down the left side and two columns for each slide number. One column is labeled “Best” and one column is labeled “Worst.”

For each slide, please think about which of the five features is the best one and which of the five features is the worst one.

Keep in mind that sometimes all five features could be considered bad. In that case, the best feature is the one that you think is the least bad. Likewise, there could be slides on which all features could be considered good. In that case, the worst feature you think is the one that is the least good.

Now, please look at the paper in front of you. Once you have identified the feature on the slide that you think is best, please put the number corresponding to that feature in the “Best” column on the line corresponding to that slide number. Likewise, once you have identified the feature on the slide that you think is worst, please put the number corresponding to that feature in the “Worst” column on the line corresponding to that slide number.

Does anyone have any questions about the task we have asked you to do?

**[Answer participant questions to ensure that participants understand the task.]**

There are 11 more slides like this. For each slide, let’s do the same thing you did for the first slide. Let’s begin.

**[Present each slide and ask participants to record the best and worst for each slide until all slides have been presented.]**

Thank you. Please pass your papers back to me.

Summing Up (~10 minutes)

Thank you for sharing your thoughts and experiences with us today. Is there anything related to your current anemia treatment that you would like to share with us?

**[Discuss issues raised.]**

Your input has been very helpful to us. Thank you again!

**SURVEY INSTRUMENT**

Dialysis Survey

Thank you for agreeing to take this survey about dialysis. First, we would like to ask you a few questions about your experience with dialysis.

1. How long have you been receiving dialysis?

- Less than 6 months
- 6 months to less than 1 year
- 1 year to less than 2 years
- 2 years to less than 5 years
- 5 years to less than 10 years
- 10 years or more

1. Have you previously received a kidney transplant?

- Yes
- No

1. Are you on a kidney transplant waiting list?

- I am currently on a kidney transplant waiting list
- I am in the process of getting on a kidney transplant waiting list
- I am not on a kidney transplant waiting list

1. Which of the following problems have you ever experienced because of your kidney disease? *(Check all that apply)*

- Bleeding in the stomach or intestines
- Bone, joint, or muscle pain
- Muscle weakness
- Weakening bones or bone fractures
- Changes in blood sugar (glucose)
- Fluid buildup in the lungs
- Hepatitis B, hepatitis C, or liver failure
- High blood pressure, heart attack, or heart failure
- High potassium levels
- Lack of appetite or poor nutrition
- Nerve damage or nervous system problems (such as restless legs syndrome)
- Secondary hyperparathyroidism (high levels of parathyroid hormone, also called *SHPT*)
- Seizures
- Skin infection
- Stroke
- Swelling or edema
- None of the above

Anemia

People who are on dialysis often develop anemia. Anemia occurs when there are not enough red blood cells in your blood. Red blood cells contain hemoglobin, which carries oxygen throughout your body. When your hemoglobin level is too low, you may not feel well.

If you have anemia for a long time, you can develop heart disease, which can increase your risk of dying.

When you have anemia, you may:

| - Feel tired and have little energy for your daily activities - Have a rapid heartbeat - Have little or no appetite - Feel depressed or “down in the dumps” - Have trouble thinking clearly - Feel dizzy or have headaches - Feel short of breath - Have trouble sleeping - Look pale |  |
| --- | --- |

The main goals for treating anemia are to:

- Help you feel better by relieving your anemia symptoms, and
- Reduce the need for blood transfusions

Some of the medicines we will ask you to think about help you feel better by relieving your anemia symptoms but do not reduce the need for blood transfusions. Others reduce your need for blood transfusions but do not help you feel better. Some medicines do both.

Your Experience With Anemia

1. Have you ever been told by a doctor or other health care professional that you have anemia?

- Yes
- No (Skip to next section)
- Don’t know / not sure (Skip to next section)

1. Do you currently have anemia?

- Yes
- No

1. Which of the following symptoms of anemia have you ever experienced? *(Check all that apply)*

- Feel tired and have little energy for your daily activities
- Have a rapid heartbeat
- Have little or no appetite
- Feel depressed or “down in the dumps”
- Have trouble thinking clearly
- Feel dizzy or have headaches
- Feel short of breath
- Have trouble sleeping
- Look pale
- None of the above

Features of Anemia Medicines

The next few pages of this survey will describe features of different anemia medicines. This information will help you answer questions later in the survey. You can refer back to this information as you take the survey.

Even if you currently don’t have anemia or if you have never been told by your doctor that you had anemia, we are still interested in your opinions about features of anemia medicine.

Medicine Feature: Chance That the Medicine Makes You Feel Better by Relieving Your Anemia Symptoms

Anemia medicines may help relieve your anemia symptoms. Relieving these symptoms helps you feel better.

1. Which of the following anemia symptoms would bother you the most?
   (*Check only 1 answer*)

- Feeling tired and having little energy for your daily activities
- Shortness of breath
- Trouble thinking clearly

Helping You Think About the Chance That the Medicine Makes You Feel Better by Relieving Your Anemia Symptoms

We will use pictures to help you think about how many patients will feel better because the anemia medicine relieves their anemia symptoms.

Each figure in the picture below represents 1 person who has anemia and takes an anemia medicine. There are 100 figures in the picture. The picture shows the chance that a person feels better because the medicine relieves anemia symptoms. The figures shown in color indicate people who **would** feel better because the medicine relieves their symptoms. The figures in gray indicate people who **would not** feel better because the medicine does not relieve their symptoms.

In the example below:

- The figures in color show that 75 people out of 100 (75%) **would** feel better because the medicine relieves their symptoms.
- The gray figures show that 25 people out of 100 (25%) **would not** feel better because the medicine does not relieve their symptoms.

When there are more figures in color, the chance of having your symptoms relieved by the medicine is higher.

Example 1:


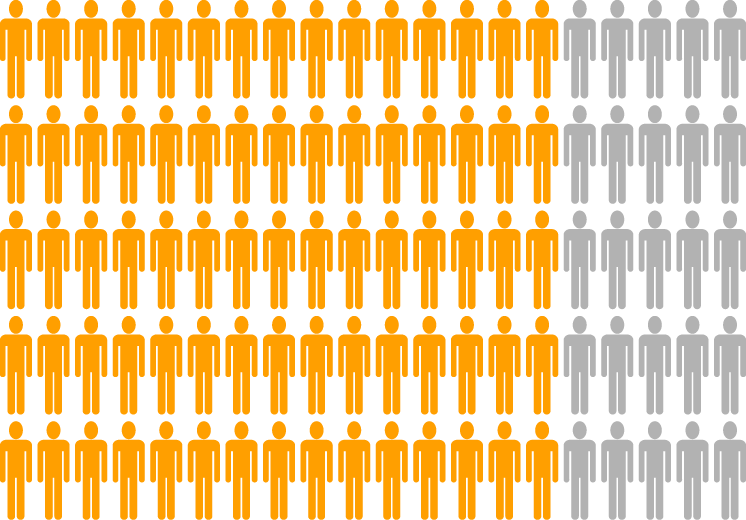


**There are 75 figures in color. That means that 75 people out of 100 (75%) who take this medicine will feel better because the medicine relieves their anemia symptoms.**

Please look at the picture below:


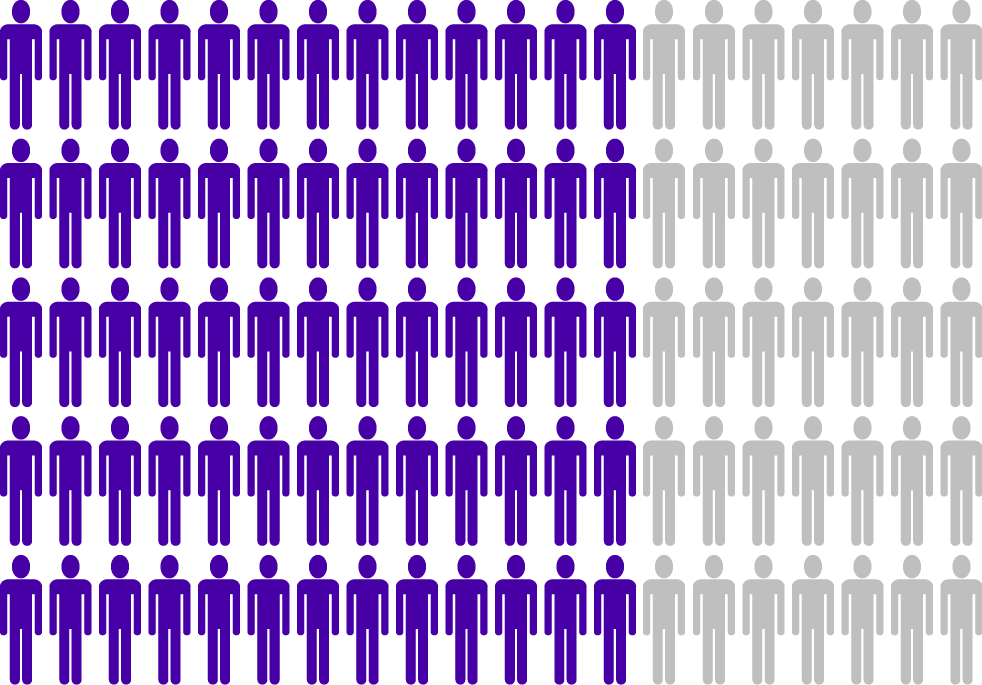


1. If each figure in the picture is 1 person who takes an anemia medicine, how many people who take the medicine would feel better because the medicine relieves their anemia symptoms?

- 90 out of 100 (90%)
- 80 out of 100 (80%)
- 65 out of 100 (65%)
- 20 out of 100 (20%)

Remember that each figure in the picture represents someone who is taking an anemia medicine. There are 100 figures in the picture. The figures in color indicate that 65 people taking a medicine to treat their anemia would feel better because the medicine relieves their anemia symptoms. Therefore, 65 out of 100 people (65%) is the answer.

Red Blood Cell Transfusions

When you are on dialysis and have anemia, you may need red blood cell transfusions. You may need a blood transfusion if you have:

- Bleeding or recently had surgery,
- Symptoms of heart disease that are made worse by anemia, or
- Severe anemia symptoms.

Red blood cell transfusions may be life-saving; however, blood transfusions have risks. Possible risks include:

- Allergic reactions
- Lung damage that makes it hard to breathe
- Serious infections
- Increasing the time you need to wait for a kidney transplant
- Increasing the chance your body will reject a kidney transplant if you get one

Red blood cell transfusions are usually given at a hospital or outpatient infusion center. Blood transfusions are not given in dialysis centers where you go for your regular dialysis treatments. This means that, if you need a blood transfusion, you will need to schedule a separate visit to a hospital or outpatient infusion center and arrange transportation. Receiving a blood transfusion usually takes 1 to 2 hours.

Please indicate whether each statement below is true or false.

|  | | | True | False | | |
| --- | --- | --- | --- | --- | --- | --- |
| 1. You may need a blood transfusion if you have severe anemia symptoms. |  | | |  |  |  |
| 1. Blood transfusions are usually done at home. |  | | |  |  |  |
| 1. You can have a blood transfusion at the same time that you are getting your dialysis treatment. |  | | |  |  |  |

Medicine Feature: Number of Red Blood Cell Transfusions Needed Each Month

One goal of anemia medicines is to reduce the need for red blood cell transfusions. If you take an anemia medicine, you may need blood transfusions less often. Some people who take an anemia medicine will not need any blood transfusions. Some people who take an anemia medicine may need 1 or 2 blood transfusions each month.

1. Have you ever had a red blood cell transfusion?

- Yes
- No
- Don’t know / not sure

1. How long ago was your most recent red blood cell transfusion?

- Less than 1 month ago
- At least 1 month ago, but less than 2 months ago
- At least 2 months ago, but less than 6 months ago
- At least 6 months ago to 1 year ago
- More than 1 year ago

1. How many times have you received a red blood cell transfusion in the past year?

- None
- 1
- 2
- 3
- 4 or more

1. Suppose you have anemia and your doctor tells you that you need to take a new anemia medicine. Also suppose that there are 2 hypothetical medicines you can take, Medicine A and Medicine B.

- 50 out of 100 people (50%) who take Medicine A will feel better because the medicine relieves their anemia symptoms, and the people who take Medicine A will not need to have blood transfusions.
- 75 out of 100 people (75%) who take Medicine B will feel better because the medicine relieves their anemia symptoms, but each person who takes Medicine B will need to have 2 blood transfusions each month.

Please indicate which medicine you would choose.

| Medicine Feature | Medicine A | Medicine B |
| --- | --- | --- |
| **Chance that the medicine makes you feel better by relieving your anemia symptoms** | 50 out of 100 (50%)  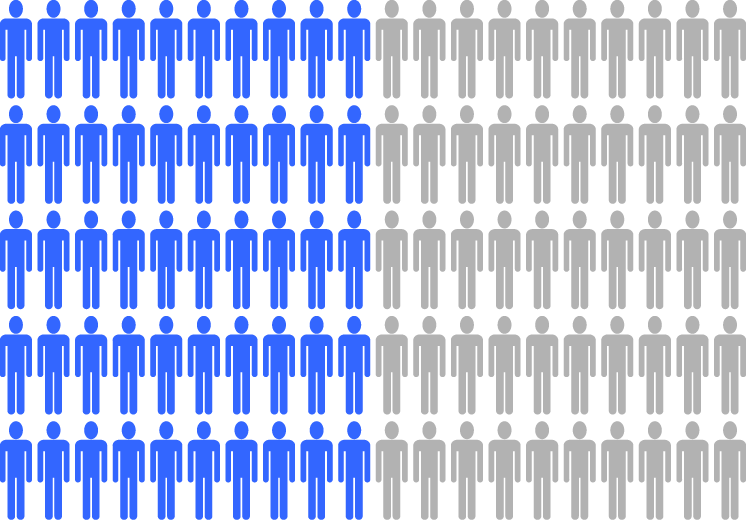 | 75 out of 100 (75%)  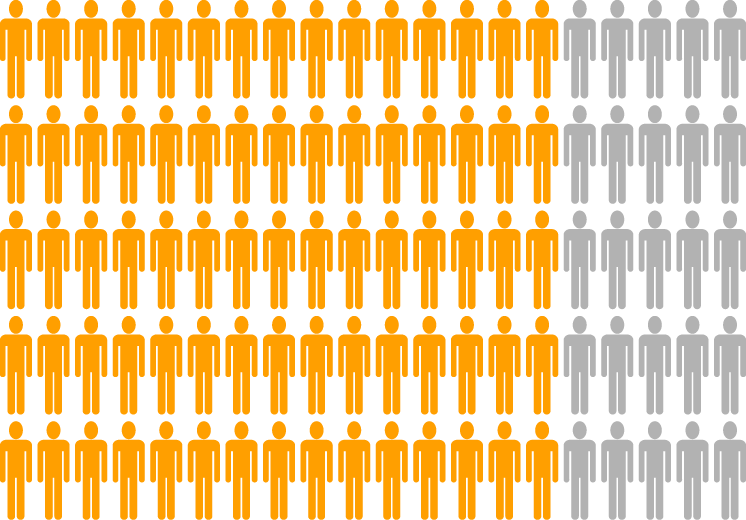 |
| **Number of red blood cell transfusions needed each month** | **0** transfusions each month | **2** transfusions each month |
|  |  |  |
| **Which medicine would you choose?** |  |  |

Medicine Feature: Risk of Dying From a Heart Attack or Stroke Because of the Medicine

Some anemia medicines may increase the chance you can have a problem such as a major heart attack or major stroke, which could be fatal.

- A major heart attack causes severe damage to the heart that cannot be treated.
- A major stroke causes severe damage to the brain that cannot be treated.

A major heart attack or stroke can cause death instantly or within a few hours.

1. Have you ever experienced a heart attack or stroke?

- Yes
- No
- Don’t know / not sure

Thinking About the Risk of Dying From a Heart Attack or Stroke Because of the Medicine

We will use pictures to help you think about how many patients will die from a heart attack or stroke because of the medicine.

Each figure in the picture below represents 1 person who receives an anemia medicine. There are 100 figures in the picture.

- The figures shown in color indicate people who **will** die from a heart attack or stroke because of the medicine.
- The figures in gray indicate people who **will not** die from a heart attack or stroke because of the medicine.

When there are more figures in color, your risk of dying from a heart attack or stroke because of the medicine is higher.

Example 1:


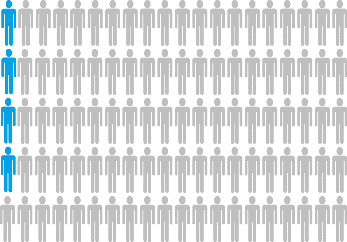


**There are 4 figures in color. That means that 4 people out of 100 (4%) who take this medicine will die from a heart attack or stroke because of the medicine.**

Please look at the picture below:


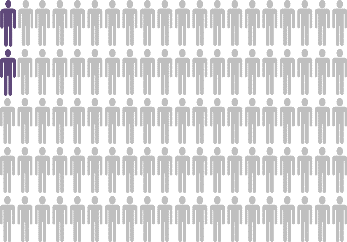


1. If each figure in the picture is 1 person who takes an anemia medicine, how many people who take the medicine will die from a heart attack or stroke because of the medicine?

- 1 out of 100 (1%)
- 2 out of 100 (2%)
- 4 out of 100 (4%)
- 98 out of 100 (98%)

Remember that each figure in the picture represents someone who is taking an anemia medicine. There are 100 figures in the picture. The figures in color indicate that 2 people taking a medicine to treat their anemia will die from a heart attack or stroke because of the medicine. Therefore, 2 out of 100 people (2%) is the correct answer.

Medicine Feature: How You Receive the Medicine

Anemia medicines are given as part of your regular dialysis treatment. There are two ways you could receive an anemia medicine:

- As an injection directly into the dialysis line during your regular dialysis treatment
- As an injection under your skin while you are receiving your regular dialysis treatment

1. How do you usually receive your anemia medicine?

- As an injection directly into the dialysis line during my regular dialysis treatment
- As an injection under my skin while I am receiving my regular dialysis treatment
- I do not currently take an anemia medicine

Thinking About Anemia Medicines

1. Again, suppose you have anemia and your doctor tells you that you need to take a new anemia medicine. Also suppose that there are 2 hypothetical medicines you can take, Medicine A and Medicine B.

| Medicine Feature | Medicine A | Medicine B |
| --- | --- | --- |
| **Chance that the medicine relieves your anemia symptoms** | 50 out of 100 (50%)  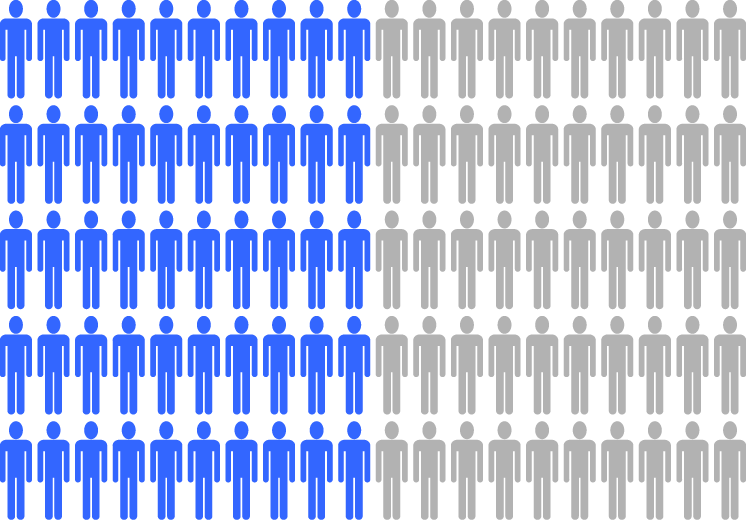 | 75 out of 100 (75%)  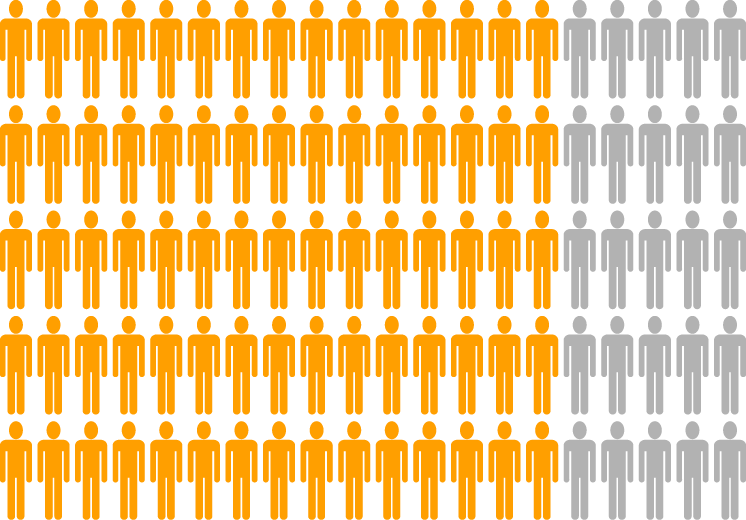 |
| **Number of red blood cell transfusions needed each month** | **1** transfusion each month | **0** transfusions each month |
| **Risk of dying from a heart attack or stroke because of the medicine** | 0 out of 100 (0%)  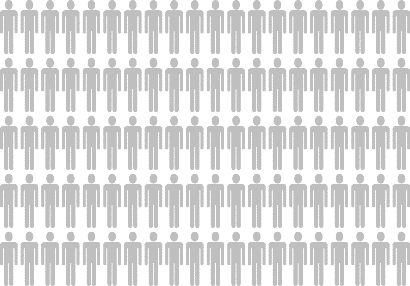 | 2 out of 100 (2%)  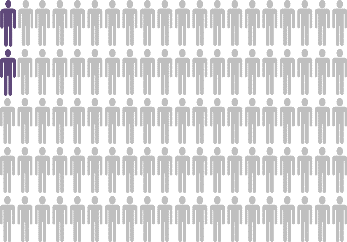 |
| **How you receive the medicine** | An injection directly into the dialysis line | An injection under your skin |
|  |  |  |
| **Which medicine would you choose?** |  |  |

Medicine Feature: Out-of-Pocket Cost Each Month

We will ask you to think about the costs of anemia medicines. Medicine costs refer to what you personally would pay out of your own pocket each month, not what your health plan or insurance company would pay.

1. About how much do you personally pay for your anemia medicine each month?

- Nothing
- $50 or less
- $51 to $100
- $101 to $250
- $251 to $500
- More than $500
- Don’t know / not sure
- I do not take an anemia medicine

1. About how much do you personally pay for all your prescription medicines each month?

- Nothing
- $50 or less
- $51 to $100
- $101 to $250
- $251 to $500
- More than $500
- Don’t know / not sure

Your Opinions About Anemia Medicines

In the next 8 questions, we will show you different pairs of possible anemia medicines your doctor might suggest. For each pair of medicines, please choose the medicine you most prefer. There are no right or wrong answers.

PLEASE HELP US

Before you tell us which medicine you would choose in each of the following questions, we need your help with a problem we have in studies like this one. People often do not think much about the costs shown in the survey because they do not really have to pay the costs. They do not think about whether the features of an anemia medicine would be worth the cost.

For example, if the costs are $50, $100, and $500, people often think of them as just "low," "medium," and "high." They do not really think about what they would have to give up—such as a restaurant meal or some new clothes—if they paid the costs.

The results of this study will not be used to set cost levels but will help us understand the value of possible anemia medicines. If you do not pay attention to the costs in each question, our results will be wrong. We will not get a true measure of how important the medicine features are.

Please help us understand your opinions by paying close attention to the cost of the medicine shown before deciding which medicine you prefer.

| Medicine Feature | Medicine A | Medicine B |
| --- | --- | --- |
| **Chance that the medicine makes you feel better by relieving your anemia symptoms** | 75 out of 100 (75%)  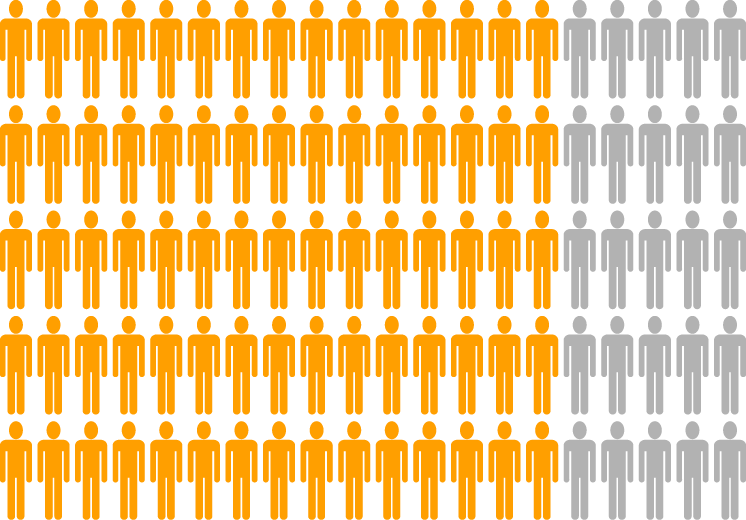 | 50 out of 100 (50%)  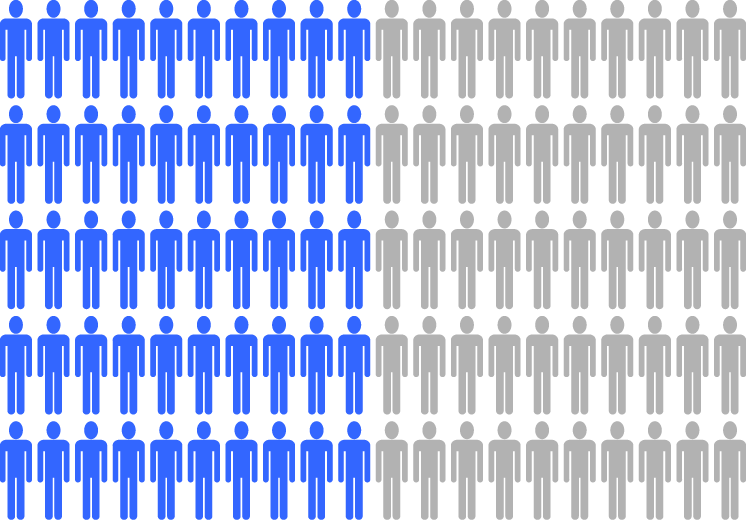 |
| **Number of red blood cell transfusions needed each month** | **1** transfusion each month | **0** transfusions each month |
| **Risk of dying from a heart attack or stroke because of the medicine** | 2 out of 100 (2%)  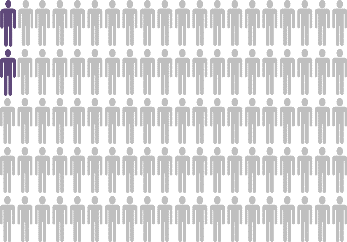 | 2 out of 100 (2%)  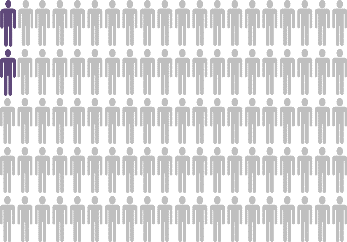 |
| **How you receive the medicine** | An injection directly  into the dialysis line | An injection  under your skin |
| **Out-of-pocket cost each month** | $100 each month | $100 each month |
|  |  |  |
| **Which medicine would you choose?** |  |  |

Other Features of Red Blood Cell Transfusions and Anemia Medicines

There are many features of red blood cell transfusions and anemia medicines that were not included in the questions you just answered.

We are interested in knowing how bothered you would be by each of these possible features of red blood cell transfusions and anemia medicines.

The table below shows 3 possible features of red blood cell transfusions or anemia medicines. Please tell us which one would bother you the most. In other words, which one would you want to avoid the most?

| I would be  bothered by this  the MOST (Check only ONE) | Features |
| --- | --- |
|  | Increasing the chance your body will reject a kidney transplant if you get one because of a blood transfusion |
|  | Needing to arrange transportation and spend 1 to 2 hours at a hospital or infusion center to receive a blood transfusion |
|  | Having a 1% risk of dying from a heart attack or stroke because of the anemia medicine |

Now, please tell us which of these same features bothers you the least. In other words, which one would you choose if you had to experience one of these features?

| Features | I would be  bothered by this  the LEAST (Check only ONE) |
| --- | --- |
| Increasing the chance your body will reject a kidney transplant if you get one because of a blood transfusion |  |
| Needing to arrange transportation and spend 1 to 2 hours at a hospital or infusion center to receive a blood transfusion |  |
| Having a 1% risk of dying from a heart attack or stroke because of the anemia medicine |  |

In each of the next 6 questions, we will show you a set of 3 features of red blood cell transfusions or anemia medicines. For each set of features, please select the feature that would bother you the most by checking the box to the left of the feature.

Then, please select the feature that would bother you the least by checking the box to the right of that feature.

Please choose only one feature as the most bothersome and one feature as the least bothersome.

| I would be  bothered by this  the MOST (Check only ONE) | Medicine Features | I would be  bothered by this  the LEAST (Check only ONE) |
| --- | --- | --- |
|  | Having an allergic reaction because of a blood transfusion |  |
|  | Increasing the time you need to wait for a kidney transplant because of a blood transfusion |  |
|  | Increasing the chance your body will reject a kidney transplant if you get one because of a blood transfusion |  |

| I would be  bothered by this  the MOST (Check only ONE) | Features | I would be  bothered by this  the LEAST (Check only ONE) |
| --- | --- | --- |
|  | Getting a serious infection because of a blood transfusion |  |
|  | Having lung damage because of a blood transfusion that makes it hard to breathe |  |
|  | Increasing the chance your body will reject a kidney transplant if you get one because of a blood transfusion |  |


| I would be  bothered by this  the MOST (Check only ONE) | Features | I would be  bothered by this  the LEAST (Check only ONE) |
| --- | --- | --- |
|  | Having lung damage because of a blood transfusion that makes it hard to breathe |  |
|  | Having an allergic reaction because of a blood transfusion |  |
|  | Needing to arrange transportation and spend 1 to 2 hours at a hospital or infusion center to receive a blood transfusion |  |

| I would be  bothered by this  the MOST (Check only ONE) | Features | I would be  bothered by this  the LEAST (Check only ONE) |
| --- | --- | --- |
|  | Having a 1% risk of dying from a heart attack or stroke because of the anemia medicine |  |
|  | Increasing the time you need to wait for a kidney transplant because of a blood transfusion |  |
|  | Having lung damage because of a blood transfusion that makes it hard to breathe |  |

| I would be  bothered by this  the MOST (Check only ONE) | Features | I would be  bothered by this  the LEAST (Check only ONE) |
| --- | --- | --- |
|  | Needing to arrange transportation and spend 1 to 2 hours at a hospital or infusion center to receive a blood transfusion |  |
|  | Getting a serious infection because of a blood transfusion |  |
|  | Increasing the time you need to wait for a kidney transplant because of a blood transfusion |  |

| I would be  bothered by this  the MOST (Check only ONE) | Features | I would be  bothered by this  the LEAST (Check only ONE) |
| --- | --- | --- |
|  | Getting a serious infection because of a blood transfusion |  |
|  | Having an allergic reaction because of a blood transfusion |  |
|  | Having a 1% risk of dying from a heart attack or stroke because of the anemia medicine |  |

Other Questions About You

1. In what year were you born? ___________
2. What is your gender?

- Female
- Male
- Prefer not to answer

1. How would you describe your race or ethnicity? (*Check all that apply*)

- White or Caucasian
- Black or African American
- Asian
- Hispanic or Latino
- Native Hawaiian or Other Pacific Islander
- American Indian or Alaska Native
- Other
- Prefer not to answer

1. What is your marital status?

- Single / never married
- Married / living as married / civil partnership
- Divorced or separated
- Widowed / surviving partner
- Other
- Prefer not to answer

1. What is the highest level of education you have completed?

- Less than high school
- Some high school
- High school or equivalent (e.g., GED)
- Some college but no degree
- Technical school
- Associate’s degree (2-year college degree)
- 4-year college degree (e.g., BA, BS)
- Some graduate school but no degree
- Graduate or professional degree (e.g., MBA, MS, MD, PhD)

1. Which of the following best describes your employment status?

- Employed full-time
- Employed part-time
- Self-employed
- Homemaker
- Student
- Retired
- Disabled / unable to work
- On medical leave of absence from work
- Unemployed but looking for work
- Unemployed and not looking for work

1. What type of health insurance do you have? (*Check all that apply.*)

- I do not have health insurance
- Private insurance that I pay for myself
- Private insurance that my or my spouse’s employer pays all or part of
- Medicaid
- Medicare
- Veterans Health insurance
- Other
- Don’t know / not sure
- Prefer not to answer

1. What was your total household income before tax and other deductions in 2014?

- Less than $20,000
- $20,000 to $29,999
- $30,000 to $39,999
- $40,000 to $49,999
- $50,000 to $59,999
- $60,000 to $69,999
- $70,000 to $79,999
- $80,000 to $89,999
- $90,000 to $99,999
- $100,000 to $149,999
- $150,000 to $199,999
- $200,000 or more
- Don’t know / not sure
- Prefer not to answer
